# Westernisation of Alcohol Consumption in Women: A Rapid Pathway to Dependence and Increased Detoxification Service Use

**DOI:** 10.1192/j.eurpsy.2025.946

**Published:** 2025-08-26

**Authors:** Â. Ferreira, I. Pereira, M. Cameira, M. Andrade, S. Morais, J. Teixeira

**Affiliations:** 1 São Bernardo Hospital, Setúbal; 2 Júlio de Matos Hospital, Lisbon; 3 Garcia de Orta Hospital, Almada, Portugal

## Abstract

**Introduction:**

The Westernisation of alcohol consumption habits among women has led to increased alcohol intake and a rise in Alcohol Use Disorder (AUD) cases in females. Although AUD prevalence remains higher in men (7.7% compared to 1.5% in women), women display distinct features in the disorder’s progression, such as the telescoping effect, where the transition from initial consumption to dependence occurs more rapidly. This trend underscores the importance of studying frequent female users of alcohol detoxification services to identify contributing factors to this accelerated progression.

**Objectives:**

This study seeks to characterise frequent female users of alcohol detoxification services, focusing on gender differences, particularly the faster progression of alcohol dependence in women and the influence of Westernisation on their drinking habits.

**Methods:**

A retrospective analysis was performed on clinical records from 2022 to 2023 for patients hospitalised at the Alcohol Treatment and Rehabilitation Unit. The analysis included patients who were hospitalised two or more times within a 12-month period. Demographic, psychosocial, and clinical variables were compared, with a particular focus on female patients.

**Results:**

Of the 360 patients admitted between 2022 and 2023, 37 were readmitted within 12 months. Women constituted 29.7% of these cases, with an average age of 52 years (compared to 51 in men), and were more often divorced or separated (81.8% vs 76.9%) and unemployed (72.7% vs 69.2%). Psychiatric family history (72.3% vs 65.4%) and psychiatric comorbidities (90.9% vs 61.5%) were more prevalent in women. Women also had a higher total number of hospitalisations (4.09 vs 3.62), although their 12-month readmission rates were similar to men (1.46 vs 1.50).

**Conclusions:**

These findings support the literature on the telescoping effect, evidenced by women’s older age and a similar number of readmissions despite a more precarious social situation and greater psychiatric comorbidities. Notably, although women were a minority among readmissions, their proportion exceeded the expected prevalence rate (30%/70% vs 15%/85%). This highlights the necessity of personalised therapeutic approaches that address the unique factors perpetuating AUD in women.

**Disclosure of Interest:**

None Declared

